# Association of cardiovascular risk factors with the different presentations of acute coronary syndrome^1^

**DOI:** 10.1590/0104-1169.3389.2449

**Published:** 2014

**Authors:** Evelise Helena Fadini Reis Brunori, Camila Takáo Lopes, Agueda Maria Ruiz Zimmer Cavalcante, Vinicius Batista Santos, Juliana de Lima Lopes, Alba Lucia Bottura Leite de Barros

**Affiliations:** 2 Doctoral students, Escola Paulista de Enfermagem, Universidade Federal de São Paulo, São Paulo, SP, Brazil; 3 PhD, Adjunct Professor, Escola Paulista de Enfermagem, Universidade Federal de São Paulo, São Paulo, SP, Brazil; 4 PhD, Full Professor, Escola Paulista de Enfermagem, Universidade Federal de São Paulo, São Paulo, SP, Brazil

**Keywords:** Acute Coronary Syndrome, Risk Factors, Myocardial Infarction, Angina Pectoris

## Abstract

**OBJECTIVE:**

to identify the relationship between different presentations of acute coronary syndrome and cardiovascular risk factors among hospitalized individuals.

**METHOD:**

cross-sectional study performed in a teaching hospital in São Paulo, in the State of São Paulo (SP). Socio-demographic, clinical and anthropometric data of 150 individuals hospitalized due to acute coronary syndrome were collected through interviews and review of clinical charts. Association between these data and the presentation of the syndrome were investigated.

**RESULTS:**

there was a predominance of ST segment elevation acute myocardial infarction. There was significant association of systemic hypertension with unstable angina and high values of low density lipoprotein with infarction, without influence from socio-demographic characteristics.

**CONCLUSION:**

arterial hypertension and high levels of low-density lipoprotein were associated with different presentations of coronary syndrome. The results can provide support for health professionals for secondary prevention programs aimed at behavioural changing.

## Introduction

Cardiovascular diseases (CVD) are the main causes of morbidity and mortality in Brazil and worldwide^(1) ^and constitute a serious public health issue. In Brazil, between January and October 2012, circulatory diseases represented 20.6% of all deaths, 24% affecting adults between 20 and 59 years old, in the prime of their productive years. Death from acute myocardial infarction (AMI) represented 12.1% in this group^(^ 2 ^)^.

Among the non-modifiable risk factors for the development of CVD, age over 55 years old, a family history of CVD, male sex, and ethnicity for certain conditions can be mentioned. Some of the modifiable risk factors are dyslipidemia (DLP), smoking, systemic arterial hypertension, physical inactivity, obesity, diabetes mellitus (DM), unhealthy diet and psycho-social stress. Dyslipidemia is the main predictor of CVD, mainly due to the high serum concentrations of low density lipoproteins (LDL)^(^ 3 ^)^. 

Excessive LDL levels in the circulation contribute to the formation of atheromatous plaques in the arterial endothelium, whose presence in the coronary artery progressively reduces the vessel's lumen, restricting blood flow and possibly leading to Acute Coronary Syndrome (ACS)^(^ 4 ^)^. The signs and symptoms of ACS constitute a continuum of intensity, from unstable angina (UA) to non-ST segment elevation myocardial infarction (NSTEMI) and ST segment elevation myocardial infarction (STEMI). Unstable angina and NSTEMI result from a coronary artery partially or intermittently occluded by the formation of thrombus on the plaque, whereas STEMI is the result of a coronary artery totally occluded by a thrombus^(^ 4 ^)^. 

The importance of controlling modifiable factors, such as hyperrtension, DM and DLP is reinforced by the Brazilian Society of Cardiology Guidelines, which also emphasize their role of independent markers for a worse prognosis among individuals with UA and NSTEMI^(^ 5 ^-^ 6 ^)^.

Prevention is understood as a basic pillar in the reduction of morbidity and comorbidity rates, and must be prioritized for individuals who present with risk factors for the development of ACS^(^ 7 ^)^.

In order for preventive measures to be undertaken, investigation of individual characterization according to the different presentations of ACS is necessary, because these presentations are associated with distinct outcomes in hospitalization. Percutaneous coronary intervention (PCI) is more frequent among patients with AMI than among those with UA, and coronary artery bypass grafting is more frequently performed in patients with UA^(^ 8 ^)^. Thereby, the different prevalence of risk factors in the individuals may influence the presentations of ACS and, as a consequence, patients' clinical outcome. 

Recent studies have investigated factors related to ACS according to its clinical manifestation in patients hospitalized following the first episode^(^ 8 ^-^ 9 ^)^. However, there are no studies investigating the relationship between the different presentations of ACS and cardiovascular risk factors. This information could contribute to knowledge expansion and scientific strengthening of health professionals, instrumentalizing them in the implementation of preventive actions towards the needs of the healthcare users, thereby allowing safe acting in relation to the process of re-education and transformation of this clientele. 

In this context, the aim was to identify the relationship between the different presentations of ACS and the cardiovascular risk factors among individuals hospitalized for the syndrome. 

## Methods

This descriptive cross-sectional study was performed in the Coronary Care Unit and Cardiology Ward of a large hospital, *Hospital São Paulo*, the teaching hospital of the Federal University of São Paulo. 

The sample consisted of 150 patients hospitalized following the first ACS event, over 18 years old and literate. Patients with acute pain, dyspnea or symptomatic hypotension at the time of data collection were excluded, due to the discomfort or tiredness that they could present during the interview. 

The sample size was calculated using the Z statistical test, normal distribution, estimating a proportion referent to the population of interest for a level of significance of 5% and sample power of 90%, resulting in a minimum sample size of 138 patients. 

Data collection occurred through interviews and the review of clinical charts from September 2011 to May 2012.The instrument used for data collection was constructed by the researchers, based on classifications of the Brazilian Institute of Geography and Statistics (IBGE, in Portuguese) and on the Brazilian Guidelines for UA, NSTEMI and STEMI, and divided into the following parts: 1) Socio-demographic information: Hospital code, sex, color (White, Black, Asian, Mixed race) and age (complete years), educational level (no education, incomplete or complete Primary education, incomplete or complete Secondary education, incomplete or complete Higher Education, Postgraduation), family income (less than one minimum salary through to nine minimum salaries), marital status/family situation (single, married, divorced, widowed, living with partner); 2) Clinical variables: family antecedents (hypertension, DM, coronary artery disease, DLP, stroke, renal failure, peripheral vascular disease, comorbidities (hypertension, DM, DLP, stroke, peripheral vascular disease, congestive heart failure), medical diagnosis, cardiac catheterization results, medical treatment undertaken and results of fasting laboratory tests collected on the first day of inpatient treatment: fasting blood glucose, total cholesterol (TotCH), high density lipoprotein (HDL), LDL and triglycerides (TG); 3) Anthropometric variables: weight (Kg), height (cm), abdominal circumference (cm) and waist-hip ratio (WHR). 

All data, apart from family income, abdominal circumference and hip measurements were recorded based on the notes made in the clinical charts. Patients were asked about their family income. Abdominal circumference was measured with a tape measure at the height of the navel, with the patient supine. Hip measurement was undertaken with a tape measure at the maximum extension of the buttocks, on the horizontal plane; the tape measure was held over the skin without pressing on soft parts.

The classification of individuals according to the glycemia results was based on: normal fasting blood glucose<100mg/dL; reduced glucose tolerance: fasting blood glucose >100 and <120mg/dL; DM: fasting blood glucose ≥126mg/dL^(^ 10 ^)^.

The serum lipid values considered normal were: TotCh<200mg/dL; LDL<160mg/dL; TG≤150mg/dL; HDL>40 mg/dL for men and >50mg/dL for women^(^ 11 ^)^.

Waist-hip ratio was obtained through the division of the abdominal circumference measurement by the hip measurement. The result was assessed according to the World Health Organization (WHO) cut-off points: for men, <1 is favorable and ≥1.1 is unfavorable, and for women, <0.85 is favorable, and ≥0.85 is unfavorable^(^ 12 ^)^.

The classification for obesity was obtained according to the Body Mass Index (BMI), based on the values stipulated by WHO, in kg/m^2^: Low weight <18.5; Normal weight: 18.5 to 24.9; Overweight: 25 to 29.9; Obesity grade I: 30 to 34.9; Obesity grade II: 35 to 39.9; Obesity grade III: ≥40^(^ 13 ^)^.

It should be noted that the interview and collection of laboratory tests occurred with a maximum difference of 24 hours. 

For data processing and statistical analysis, the Statistical Package for the Social Sciences software, version 19.0, was used. The categorical variables were summarized through descriptive statistics of frequencies (absolute and relative). The numerical variables were summarized as means and standard deviation. The association between qualitative measurements and the diagnoses was assessed using Pearson's Chi-squared test or Fisher's exact test. The association between quantitative measurements and the diagnoses was assessed using Analysis of Variance or the Kruskal-Wallis test. The level of significance adopted was 5%. 

The study protocol was submitted to the Ethics Committee of the Federal University of São Paulo, protocol N. 1511/10, and approved in accordance with Resolution 196/96 of the National Health Council. Terms of consent were signed by the patients who accepted to participate in the study.

## Results

The sample consisted of 150 patients. Their socio-demographic characteristics were not significantly related to the type of presentation of ACS (Table 1). Regarding the medical diagnosis, STEMI was predominant (72.7%), followed by UA (14.7%) and NSTEMI (12.7%).

**Table 1:** Relationship of the socio-demographic characteristics with the medical diagnosis of individuals hospitalized for acute coronary syndrome. Coronary Care Unit and Ward, Hospital São Paulo. São Paulo, SP, Brazil, 2012

Variable		Medical diagnosis			Total (n=150)	p
		UA* (n=22)	NSTEMI^†^ (n=19)	STEMI^‡^ (n=109)		
Age (years)						0.184^§^
	Mean	58.41	61.63	56.61	57.51	
	Standard deviation	10.71	13.41	10.85	11.23	
Sex (%)						0.088^||^
	Female	45.5	31.6	22.9	27.3	
	Male	54.5	68.4	77.1	72.7	
Color (%)						0.943^||^
	Black	36.4	31.6	30.3	31.3	
	White	0	0	1.8	1.3	
	Asian	63.6	68.4	67.9	67.3	
Religion						0.765^¶^
	Roman Catholic	59.1	84.2	73.4	72.7	
	Evangelical	22.7	10.5	11.9	13.3	
	Spiritualist	4.5	0	3.7	3.3	
	Others	4.5	0	5.5	4.7	
	None	9.1	5.3	5.5	6.0	
Educational level (%)						0.619^¶^
	Incomplete Primary education	31.8	47.4	34.9	36.0	
	Complete Primary education	40.9	42.1	33.0	35.3	
	Incomplete Secondary education	0	5.3	7.3	6.0	
	Complete Secondary education	22.7	0	17.4	16.0	
	Incomplete Higher education	0	0	2.8	2.0	
	Complete Higher education	4.5	5.3	3.7	4.0	
	Post-graduate	0	0	0.9	0.7	
Marital status/Family situation (%)						0.518^¶^
	Single	13.6	10.5	8.3	9.3	
	Married	54.5	47.4	65.1	61.3	
	Divorced	13.6	21.1	14.7	15.3	
	Widowed	18.2	15.8	7.3	10.0	
	Lives with partner	0	5.3	4.6	4.0	
Income (minimum salaries)						0.467^¶^
	1	9.1	15.8	11.0	11.3	
	2	50.0	57.9	44.0	46.7	
	3	22.7	5.3	26.6	23.3	
	4	18.2	10.5	12.8	13.3	
	6	0	10.5	5.5	5.3	

* Unstable angina

† Non-ST segment elevation acute myocardial infarction

‡ ST segment elevation acute myocardial infarction

§ ANOVA

|| Pearson's Chi-squared test

¶ Fisher's exact test

Table 2 shows that hypertension, DLP and DM stood out among the main comorbidities in the sample and as the most prevalent family antecedents. There was a significant association of hypertension with UA (p=0.002).

**Table 2:** Relationship of the main comorbidities and family antecedents with the medical diagnosis of individuals hospitalized for acute coronary syndrome. Coronary Care Unit and Ward, Hospital São Paulo. São Paulo, SP, Brazil, 2012

		Medical diagnosis											p^§^
		UA* (n=22)			NSTEMI^†^ (n=19)			STEMI^‡^ (n=109)			Total (n=150)		
		n	%		N	%		n	%		n	%	
Comorbidity													
	Arterial hypertension	21	95.5		15	78.9		64	58.7		100	66.7	0.002
	Dyslipidemia	13	59.1		6	31.6		41	37.6		60	40.0	0.125
	Diabetes mellitus	6	27.3		7	36.8		30	27.5		43	28.7	0.701
Family antecedent													
	Arterial hypertension	14	63.6		13	68.4		79	72.5		106	70.7	0.690
	Coronary artery disease	12	54.5		10	52.6		69	63.3		91	60.7	0.555
	Dyslipidemia	9	40.9		4	21.1		32	29.4		45	30.0	0.369
	Diabetes mellitus	9	40.9		7	36.8		46	42.2		62	41.3	0.908

* Unstable angina

† Non-ST segment elevation acute myocardial infarction

‡ ST segment elevation acute myocardial infarction

§ Pearson's Chi-squared test

There was a greater frequency of patients among whom one or two coronary arteries (69.3%) were affected (Table 3). One coronary artery being affected was significantly associated with NSTEMI (p=0.029).

**Table 3:** Relationship of the number of coronary arteries affected by coronary artery disease and treatment, with the medical diagnosis of individuals hospitalized for acute coronary syndrome. Coronary Care Unit and Ward, Hospital São Paulo. São Paulo, SP, Brazil, 2012

		Medical diagnosis											p
		UA* (n=22)			NSTEMI^†^ (n=19)			STEMI^‡^ (n=109)			Total (n=150)		
		n	%		n	%		n	%		n	%	
Number of arteries													
	One artery	4	18.2		11	57.9		38	34.9		53	35.3	0.029^§^
	Two arteries	10	45.5		3	15.8		38	34.9		51	34.0	0.127^§^
	Three arteries	8	36.4		5	26.3		30	27.5		43	28.7	0.684^§^
	Trunk of left coronary artery	2	9.1		0	0		1	0.9		3	2.0	0.097^||^
Treatment													
	Clinical	12	54.5		7	36.8		1	0.9		20	13.3	<0.001^||^
	Percutaneous coronary intervention – anterior descending artery	3	13.6		5	26.3		57	52.3		65	43.3	0.001^§^
	Percutaneous coronary intervention – circumflex artery	0	0		2	10.5		14	12.8		16	10.7	0.219^||^
	Percutaneous coronary intervention – right coronary artery	0	0		1	5.3		23	21.1		24	16.0	0.011^||^
	Percutaneous coronary intervention – trunk of left coronary artery	0	0		0	0		1	0.9		1	0.7	1.000^||^
	Myocardial revascularization surgery	7	31.8		4	21.1		18	16.5		19	19.3	0.223^||^

* Unstable angina

† Non-ST segment elevation acute myocardial infarction

‡ ST segment elevation acute myocardial infarction

|| Fisher's exact test

§ Pearson's Chi-squared test

The most prevalent treatment was PCI. A significantly greater proportion of patients with UA received clinical treatment compared with that of individuals with AMI (p<0.001). Percutaneous Coronary Intervention of the anterior descending artery (ADA) and right coronary artery (RCA) was greater among those with a diagnosis of STEMI (p=0.001 and 0.0011, respectively).

As shown in Table 4, the mean values for fasting blood glucose were above the normal limits in all the participants in the study, and the values for TotCh, LDL and TG were close to the limits considered normal or above them, whereas the mean HDL value was low. In relation to WHR, all the women participating were above the normal levels. The minimum WHR for the women was 0.85. Among the men, 66% had an appropriate WHR, with a minimum value of 0.78 and a maximum value of 1.38. There was a significant association between LDL values and the diagnosis of AMI (p=0.009).

**Table 4:** Relationship of laboratory parameters and anthropometric variables with the medical diagnoses of individuals hospitalized due to acute coronary syndrome. Coronary Care Unit and Ward, Hospital São Paulo. São Paulo, SP, Brazil, 2012

Parameter	Medical diagnosis (mean ± standard deviation)				p
	UA* (n=22)	NSTEMI^†^ (n=19)	STEMI^‡^ (n=109)	Total (n=150)	
Blood glucose (mg/dL)	142.5±59.54	141.84±95.38	145.39±67.09	144.5±69.81	0.149^§^
Total cholesterol (mg/dL)	173.16±59.61	198.75±39.7	202.58±56.27	198.21±55.68	0.105^||^
High Density Lipoprotein – HDL (mg/dL)	38.21±12.07	38.69±9.92	40.99±11.72	40.36±11.56	0.523^||^
Low density lipoprotein – LDL (mg/dL)	92.22±48.67	130.38±38.09	127.98±46.3	123.69±47.04	0.009^||^
Triglycerides (mg/dL)	196.58±124.87	150.56±87.19	148.19±77.14	154.93±86.85	0.274^§^
Body Mass Index – BMI (kg/m^2^)	28.23±4.65	25.68±3.39	26.83±4.39	26.89±4.34	0.122^§^
Waist-hip ratio	0.98±0.14	0.96±0.09	0.99±0.09	0.98±0.1	0.263^§^

* Unstable angina

† Non-ST segment elevation acute myocardial infarction

‡ ST segment elevation acute myocardial infarction

§ Kruskal-Wallis test

|| ANOVA

The majority of patients were overweight (44.6%), followed by normal BMI (33.3%), obese (21.4%) and low weight (0.7%). The prevalence of grade II obesity among the women (7.3%), twice that of the men (3.7%), stands out. However, there was no association of BMI with the type of presentation of ACS. 

## Discussion

Strategic planning of CVD risk factor control which has an effective impact on a population cannot be undertaken based on generalities, and depends on prior knowledge of the population's specific characteristics. It was in this context that the relationship between the different presentations of ACS and cardiovascular risk factors, and the treatment among individuals hospitalized because of the syndrome, was investigated. 

The socio-demographic profile found is corroborated by other Brazilian studies, which found the occurrence of risk factors for coronary artery disease among individuals with ACS at a mean age of 55-60 years old, predominantly male, white, married, with incomplete Primary education and a monthly income of two to three minimum salaries^(^ 14 ^-^ 16 ^)^. These characteristics are associated with the evolution of ACS and mortality in the population affected. 

Studies show that the age range predominantly affected is a reflection of the process of development of the atherosclerotic plaque on the coronary artery wall, as the early fibroatheroma begins in adolescence and during the second decade of life, continuing throughout life. The advanced atheroma occurs in people aged over 55 years old. In this stage, a thin fibrous cap develops through proteolytic enzymatic action, which may break, exposing the thrombogenic arterial wall and producing thrombosis^(^ 17 ^)^.

Regarding the population's profile of comorbidities, data were similar to those from different studies undertaken since the 1990s, in which there occur hypertension, DM and family antecedents for coronary artery disease and cardiac insufficiency. It should be noticed that, in spite of such risk factors occurring in different samples of the Brazilian population, these data continue to rise progressively, evidencing the urgent need for aggressive combat against the risk factors already established for CVD^(^ 14 ^-^ 16 ^,^ 18 ^)^.

Concerning the different presentations of ACS, distinct studies show a profile which is similar to the findings of the present study. A retrospective cohort performed in a teaching hospital of a university in Rio de Janeiro ascertained a greater prevalence of hospitalization of individuals with STEMI (37.1%) compared with those with UA (35.6%) and NSTEMI (24.9%). Furthermore, one study performed in the non-metropolitan region of the state of São Paulo with 234 patients hospitalized for the first time due to the manifestation of ACS observed that 114 (59.8%) were diagnosed with AMI and 94 (40.2%) with UA^(^ 8 ^)^.

The same study mentioned above performed in the non-metropolitan region of São Paulo verified that the length of inpatient treatment is greater among individuals admitted with UA, although the incidence of complications is greater among those who had infarction^(^ 8 ^)^. These data are relevant because they demonstrate the importance of knowing the manifestations of the different presentations of ACS, as these show varying rates of complications and mortality. 

In the present study, women predominated among the individuals diagnosed with UA. It is believed that the persistence of symptoms of myocardial ischemia eventually led them to seek assistance, avoiding further progression of the disease. Compared with patients being hospitalized for the first time with a diagnosis of AMI, individuals with UA recognized at an earlier stage that they must seek professional help, as they present greater limitations for day-to-day activities in the week that precedes hospitalization due to ACS^(^ 19 ^)^.

Among the individuals diagnosed with NSTEMI, there was predominance of occlusion of one coronary artery. This fact may have influenced the predominant choice of clinical treatment. Among the individuals diagnosed with UA, those with occlusion of three arteries predominated. In addition, the majority of those who presented a lesion on the trunk of the left coronary artery were diagnosed with UA, which was reflected in the surgical treatment instituted for this group. 

Regarding the arterial occlusion, particular attention must be given to patients with RCA occlusion, as this artery is generally responsible for the blood supply to most of the right ventricle. Mortality from right ventricle infarction is high when accompanied by lower wall infarction (25% to 30%). Thus, these patients are considered high priority for early reperfusion^(^ 6 ^)^.

The fact that PCI was the most frequently used treatment in this study's population is a reflection of the predominance of STEMI as a manifestation of coronary artery disease, with a greater proportion of occlusion of up to two coronary arteries, mainly the ADA and RCA^(^ 14 ^)^. The type of coronary artery affected is directly related to the treatment instituted. These data may also be influenced by the time of arrival of the patients with AMI in the emergency department, which should be less than 90 minutes, this limit being stipulated by the Brazilian Society of Cardiology for PCI in this diagnosis^(^ 6 ^)^. It was recently demonstrated by nurses from the non-metropolitan region of the State of São Paulo that the arrival time of patients with infarction in a specialized emergency service varied by up to 183.3%. The minimum time for the occurrence of the event and the attendance was nine hours 45 minutes and the maximum time was 19 hours and nine minutes^(^ 20 ^)^.

In relation to the laboratory values, the presence of mean values for glycemia above normal limits in all the participants in the study was observed. The values for TotCh, LDL and TG were close to the limits considered normal or above them, whereas the mean value for HDL was low. 

Previous studies with coronary patients also found similar results to those evidenced in the samples studied regarding the presence of high levels of blood glucose, TotCh, LDL and TG and low levels of HDL^(^ 14 ^-^ 15 ^,^ 18 ^)^.

The INTERHEART study ascertained that DLP is among the most important risk factors for the occurrence of AMI^(^ 21 ^)^. In Brazil^(^ 14 ^)^ and in the metropolitan region of São Paulo^(^ 15 ^)^, the main risk factors for AMI are DM, increased WHR, a family history of coronary artery disease, increased LDL, hypertension and smoking. 

In this study, the majority of the patients had high levels of blood glucose. AMI was most frequent in patients with high levels of LDL, confirming the high prevalence of insulin resistance among individuals who present coronary artery disease and ACS, as well as the important association of high levels of LDL with the diagnoses of STEMI and NSTEMI. 

The higher plasma levels of LDL in the patients with AMI reflect the process of evolution of the atherosclerotic plaque in coronary artery disease^(^ 17 ^)^. The WHR also determines the individual's risk for developing CVD, as it defines the distribution of body fat. High WHR has been indicated as a predictive factor for CVD, irrespective of the BMI. Some studies show that men and women with high WHR values have a greater risk of death, syncope, ischemic myocardiopathy, glucose intolerance, and higher levels of blood pressure and serum lipids^(^ 18 ^)^.

These data are directly related to the lifestyle and health behavior acquired by the individual. Habits such as inappropriate eating, sedentarism, smoking and alcoholism have been highlighted by WHO and may be potential aggravants for the compromising of health and the occurrence of complications^(^ 22 ^)^.

The BMI values in both sexes varied. Among women, the presence of grade II obesity was twice as great as among the men. In addition, there was prevalence of patients with WHR with a mean of 0.98 and overweight (mean BMI of 26.89), which contributes to the development of chronic diseases, including coronary artery disease, hypertension, DLP, and type 2 DM, leading to a greater risk of cardiovascular complications and death^(^ 22 ^)^.

The incidence of obesity and overweight has markedly increased in recent years. In a study recently performed by the Brazilian Ministry of Health, the population of adult individuals overweight was 48.5%, the majority being among men (52.6%), whereas among women it was 44.7%. In relation to the obese adult population, the presence of obesity in 27 cities was 15.8%. Among men, obesity tripled from the age range of 18 to 24 years old to the age range of 35 to 44 years old^(^ 22 ^)^. 

In the World Health Report 2008, WHO emphasizes the importance of individual-centered care and also reports the differences between the problems addressed in the secondary and primary levels, emphasizing that there is a greater challenge in relation to primary care, as individuals must be holistically evaluated, according to their physical, emotional and social aspects. Hence, identification of this set of risks in this specific population is fundamental for interventions to be planned in the context of secondary prevention. The interventions must also cover the multiple metabolic abnormalities, which, in addition to preventing the appearance of diabetes, would avoid the development of CVD, thus reducing mortality^(^ 23 ^)^.

This study's results are limited by its transversal design, which does not allow the establishment of causal relationships between the characteristics studied and the manifestations of ACS. Also, data in clinical charts are not always adequately filled out. Future studies must adopt longitudinal, multicentric methods, with a larger sample size and follow-up period, thus enabling testing of the hypotheses established in the present study.

Another limitation refers to the data collection instrument used, which was not submitted for validation, although its variables were based on national guidelines for ACS. Future studies could submit the instrument for content and face validation by expert nurses. 

## Conclusions

The most frequent diagnosis was STEMI followed by UA and NSTEMI, with PCI being the treatment instituted for the majority of the patients. The majority of the sample were dyslipidemic and diabetic, and all were hypertensive. An important proportion of the patients had a positive family history and risk factors for CVD. The mean values for TotCh, LDL, fasting blood glucose and TG were high, whereas the mean value for HDL was low. The mean BMI corresponded to the range of overweight and WHR was above normal levels. There was a significant association of hypertension with UA and values of LDL with AMI, without influence from socio-demographic characteristics. 

Although the study was performed in only one hospital institution in the state of São Paulo, with a relatively small sample, it may reflect the reality of other states and countries, mainly because there are different studies with similar data to this one. However, it is believed that other investigations are necessary investigating the associations of the different types of ACS with the risk factors, socio-demographic profile and, moreover, the clinical symptoms, so as to offer a greater quantity of data and make new inferences possible. 

Therefore, knowing this risk profile will allow planning and prioritization of interventions associated with reducing the risk of occurrence of further coronary events. In addition, it can provide support for health professionals for effective practice in secondary prevention programs that aim to change the behavior of their users.
